# The influence of nicotine on granulocytic differentiation – Inhibition of the oxidative burst and bacterial killing and increased matrix metalloproteinase-9 release

**DOI:** 10.1186/1471-2121-9-19

**Published:** 2008-04-15

**Authors:** Minqi Xu, James E Scott, Kan-Zhi Liu, Hannah R Bishop, Diane E Renaud, Richard M Palmer, Abdel Soussi-Gounni, David A Scott

**Affiliations:** 1 Department of Oral Biology, University of Manitoba, Winnipeg, Canada; 2 Institute for Biodiagnostics, National Research Council, Winnipeg, Canada; 3 Oral Health and Systemic Disease Research Group, University of Louisville, Louisville, USA; 4 Department of Preventive Dentistry, King's College London, London, UK; 5 Department of Immunology, University of Manitoba, Winnipeg, Canada; 6 Department of Toxicology and Pharmacology, University of Louisville, Louisville, USA

## Abstract

**Background:**

Neutrophils leave the bone marrow as terminally differentiated cells, yet little is known of the influence of nicotine or other tobacco smoke components on neutrophil differentiation. Therefore, promyelocytic HL-60 cells were differentiated into neutrophils using dimethylsulfoxide in the presence and absence of nicotine (3-(1-methyl-2-pyrrolidinyl) pyridine). Differentiation was evaluated over 5 days by monitoring terminal differentiation markers (CD11b expression and formazan deposition); cell viability, growth phase, kinetics, and apoptosis; assessing cellular morphology and ultrastructure; and conformational changes to major cellular components. Key neutrophil effector functions (oxidative burst, bacterial killing, matrix metalloproteinase release) were also examined.

**Results:**

Nicotine increased the percentage of cells in late differentiation phases (metamyelocytes, banded neutrophils and segmented neutrophils) compared to DMSO alone (*p *< 0.05), but did not affect any other marker of neutrophil differentiation examined. However, nicotine exposure during differentiation suppressed the oxidative burst in HL-60 cells (*p *< 0.001); inhibited bacterial killing (*p *< 0.01); and increased the LPS-induced release of MMP-9, but not MMP-2 (*p *< 0.05). These phenomena may be α-7-acetylcholine nicotinic receptor-dependent. Furthermore, smokers exhibited an increased MMP-9 burden compared to non-smokers *in vivo *(p < 0.05).

**Conclusion:**

These findings may partially explain the known increase in susceptibility to bacterial infection and neutrophil-associated destructive inflammatory diseases in individuals chronically exposed to nicotine.

## Background

Tobacco smoking is a major cause of morbidity and mortality. The World Health Organization recently estimated that there are 1.1 billion current smokers [1]. As this is equivalent to one third of the world's entire population greater than 15 years of age, it is difficult to over-estimate the enormity of this health problem.

Smoking causes systemic neutrophilia [2]. Multiple tobacco-induced and/or exacerbated diseases, including COPD [3]; atherosclerosis and other vascular diseases [4], asthma [5], and periodontitis [6] have been associated with neutrophil influx, cytokine release, and degranulation events. Furthermore, neutrophils are key cells in combating microbial invasion, and smokers exhibit an increased risk of infectious diseases [7] including invasive pneumococcal disease [8], tuberculosis [7], meningitis, as well as exhibiting increased risk of infection with *Helicobacter pylori*, *Porphyromonas gingivalis, Legionella pneumophila *and other bacterial species [6,7].

Therefore, several studies have examined the influence of tobacco smoke, smoke extracts, and components of tobacco smoke on important aspects of neutrophil function, including neutrophil chemotaxis and recruitment [9], oxidative burst [10], bacterial killing clearance [11,12]; apoptosis [13]; and clearance of apoptotic neutrophils by macrophages [14]. Convincing evidence now exists that tobacco smoke is a key stimulator of aberrant neutrophil activation, which may predispose to several inflammatory diseases [2,6,15].

Neutrophils leave the bone marrow as terminally differentiated cells with low transcriptional activity that, without appropriate inflammatory stimuli, normally have a short life span of one to two days. Therefore, many important tobacco-induced alterations to neutrophil physiology may occur before neutrophils even reach the systemic circulation. However, there are essentially no data available on the effects of tobacco smoke or smoke products on neutrophil differentiation.

Cigarette smoke contains more than 4000 different compounds, many of which are toxic and/or bioactive. Studies that use whole smoke or smoke extracts may better reflect in vivo complexities. However, one major disadvantage of using complex mixtures of smoke components approach is that we still do not know which exact molecule(s) is responsible for the pathological changes so that a targeted therapeutic approach can be designed. Nicotine (3-(1-methyl-2-pyrrolidinyl) pyridine) is a principle, toxic component of tobacco smoke and both primary neutrophils and HL-60 cells are known to express nicotine receptors. Indeed, the numbers of nicotinic receptors expressed by human neutrophils have been reported to be increased in smokers and decline on cessation [16]. In the venous blood of tobacco smokers, nicotine concentrations are normally 0.5 × 10^-7 ^M to 5.0 × 10^-7 ^M, with arterial and tissue nicotine concentrations up to six to ten-fold or two to three-fold higher, respectively [17]. Levels of nicotine in the oral and pulmonary environments are higher still. Although multiple nicotine delivery systems, such as gum, patch, and spray, have been widely used for the therapeutic purpose of smoking cessation in clinic and daily life, the continuous intake of nicotine has been reported to hinder the functional recovery of neutrophils in smokers who were quitting cigarettes [18].

HL-60 cells are a well-characterized promyelocytic cell line obtained from an individual with acute promyelocytic leukemia and are commonly used as a model in granulocyte differentiation studies. As our initial data showed that HL-60 cells express nicotinic receptors, because nicotine is a major component of tobacco smoke that is distributed throughout the body, and because the study of individual tobacco components allows a more efficient targeting of specific pathogenic mechanisms, we determined to examine the influence of physiologically relevant nicotine concentrations (10^-7 ^to 10^-4 ^M) on HL-60 promyelocytes that were induced to differentiate into the neutrophil lineage with dimethylsulfoxide (DMSO). We report in this manuscript that while nicotine does not influence HL-60 proliferation, viability, cell cycle, ultrastructural characteristics and the expression of terminal differentiation markers, key effector functions, including superoxide generation, bacterial killing and MMP-9 release, are dysregulated.

## Results

### Expression of nicotinic receptors by HL-60 cells

**Figure 1:**
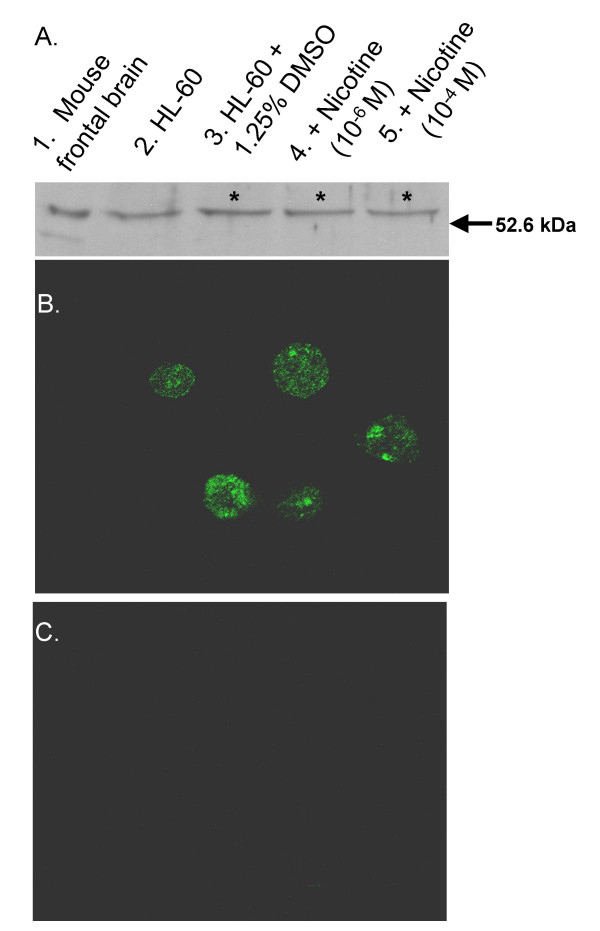
**Promyelocytic and DMSO-differentiated HL-60 cells express α_7_-acetylcholine nicotinic receptors**. **(A) **α7 nAChR Western blot: Lane 1. Mouse frontal brain extract (10 μg; positive control); Lane 2. Lysate (40 μg) of promyelocytic HL-60 cells; Lanes 3 to 5. Lysate (40 μg) of five day, DMSO-induced HL-60 cells without nicotine, or with 10^-6 ^M nicotine, or with 10^-4 ^M nicotine treatment, respectively. α7 nAChR-specific antibodies (Santa Cruz SC-5544) bound to protein bands exhibiting a relative molecular mass of 55.0 KDa in all lanes. Densitometric analysis (*data not shown*) revealed that α7 nAChR-specific band intensities were significantly increased in the DMSO-treated samples, relative to promyelocytic cells (*n *= 5, *p < 0.05), but that this differentiation-associated increase α7 nAChR protein level was not influenced by nicotine exposure during differentiation in any statistically significant manner. **(B) **α7 nAChR Immunofluorescence staining (×1000): Fixed five-day, DMSO-induced HL-60 cells were incubated with a polyclonal rabbit anti-α7 antibody (Santa Cruz SC-5544) before staining with a FITC-conjugated anti-rabbit antibody (Santa Cruz SC-2253). α7 nAChR-specific staining was evenly distributed across the cells. A similar cellular distribution of α7 nAChR was observed in promyelocytic cells (*data not shown*). **(C) **Negative control for α7 nAChR Immunofluorescence staining (×1000).

Antibodies against nicotinic acetylcholine receptor α7 subtype bound to a HL-60 protein with a 55 kDa relative molecular mass, as shown in Figure 1A. Both undifferentiated and DMSO differentiated HL-60 cells expressed the 55 kDa receptors. Differentiation with DMSO increased the expression of α7 nAChR, as determined by densitometric analysis, whereas nicotine did not. Immunofluorescence revealed that α7 nAChR appears equally distributed on the HL-60 cell surface (Figure 1B).

### Cellular morphology and ultrastructure

**Figure 2:**
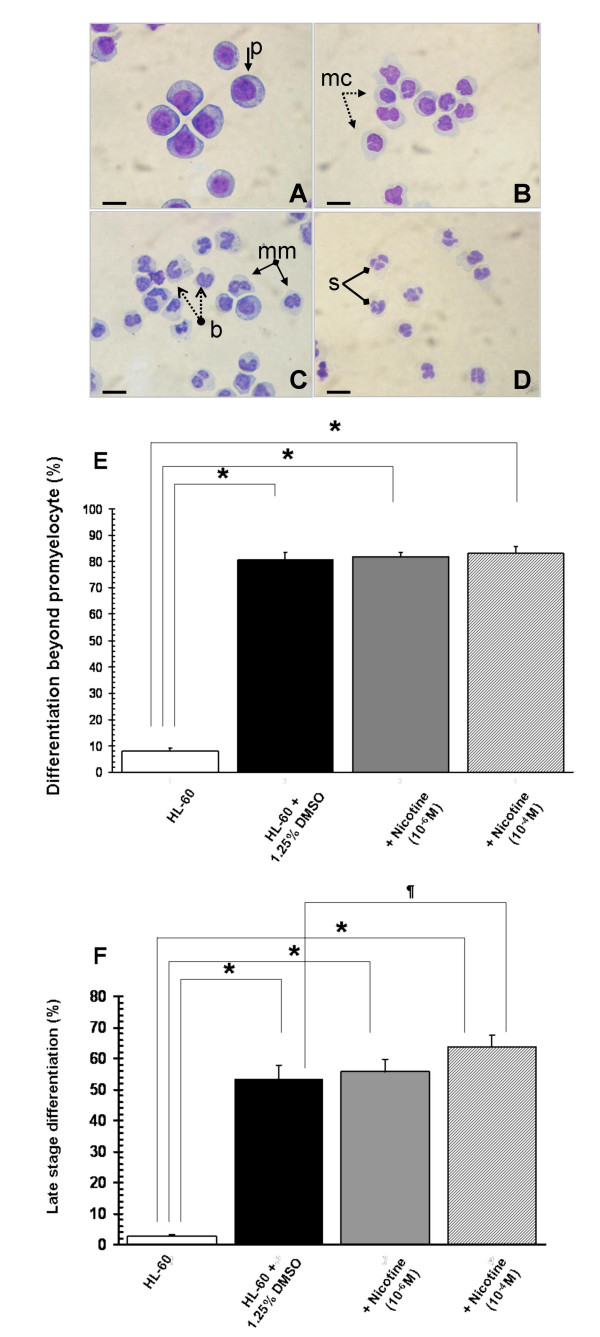
**Nicotine increases the percentage of cells reaching late stage differentiation but not other morphological characteristics of DMSO-induced HL-60 cells**. (A – D) Hema 3 staining of differentiating HL-60 cells: Promyelocytic HL-60 cells (A) were induced to differentiate 1.25% (v/v) DMSO over one (B), three (C), or five (D) days. Compared to undifferentiated HL-60 cells, the morphological characteristics of differentiating cells include reduced cell size, decreased nucleus/cytoplasma ratio, disappearance of nucleoli, transformation of nuclei, and fewer cytoplasmic granules. Images were taken on an Olympus BX41 microscopy with a magnification of 400× using a SONY 4.1 MP digital camera. p: promyelocytes; mc: myelocytes; mm: metamyelocytes; b: band cells; s: segmented neutrophil. The bars represent 10 μm. (E) DMSO exposure increased the mean percentage of cells differentiating beyond the promyelocytic stage over five-days, compared to DMSO-untreated control cells (*n *= 6, *p < 0.001); but nicotine had no statistically significant influence on the percentage of DMSO-treated HL-60 cells differentiating past the promyelocytic stage. (F) DMSO exposure increased the mean percentage of cells in late phases of differentiation (metamyelocytes, banded neutrophils and segmented neutrophils) after 5-days compared to DMSO-untreated control cells (*n *= 6, *p < 0.001); with nicotine (10^-4 ^M) exposure during DMSO-induced differentiation further increasing the percentage of late phase cells compared to DMSO alone (*n *= 6, **¶***p *< 0.05). Error bars represent standard deviations of the mean.

Myeloblasts, promyelocytes and myelocytes were considered early differentiation phases. Metamyelocytes, banded neutrophils and segmented neutrophils were considered late differentiation phases. In the absence of DMSO, HL-60 cells were predominantly typical promyelocytes with large round or oval nuclei, each containing 2–4 nucleoli and dispersed, fine nuclear chromatin. The cytoplasm was basophilic with prominent azurophilic granules. The ratio of nucleus/cytoplasm was relatively high (3:1 to 2:1). DMSO-induced HL-60 cells exhibited smaller cell size, decreased ratio of nucleus/cytoplasm, fewer prominent cytoplasmic granules, marked reduction or disappearance of nucleoli, pyknotic changes in nuclear chromatin, and notable indentation, convolution, and segmentation of the nuclei (Figure 2A–D). These morphologic data are consistent with the pioneer studies on the HL-60 cell line by Collins et al [19].

The spontaneous myeloid differentiation rate of DMSO-untreated HL-60 cells into myelocytes, metamyelocytes, band cells and mature, segmented neutrophils remained stable [7.1 ± 1.5%; (mean, s.d.)], regardless of nicotine exposure (Figure 2E). Similarly, nicotine had no statistically significant influence on the percentage of HL-60 cells differentiating past the promyelocytic stage (Figure 2E). However, nicotine (10^-4 ^M) increased the percentage of cells in late phases of differentiation compared to DMSO alone (Figure 2F).

**Figure 3:**
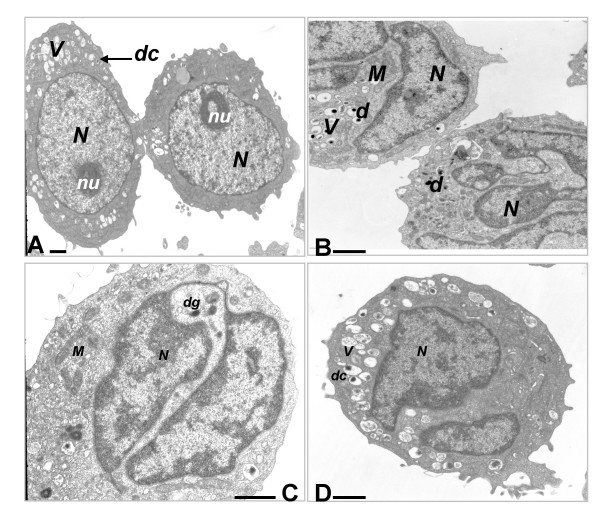
**Nicotine exposure during differentiation does not influence the ultrastructural characteristics of HL-60 cells**. Promyelocytic HL-60 cells (**A**, ×1550) contain a large or oval nucleus *(N) *with diffuse chromatin, a prominent nucleolus *(nu)*, and a variable number of large cytoplasmic vacuoles (V) some of which contain variably dense cores *(d)*; Five-day, DMSO-differentiated HL-60 cells without nicotine treatment (**B**, ×2650); or exposed to 10^-6 ^M nicotine (**C**, ×4300); or 10^-4 ^M nicotine (**D**, ×2650) contain a markedly indented and segmented nucleus with pyknotic nuclear chromatin *(N)*. The nucleolus has disappeared. Small uniformly dense granules *(dg) *as well as some large vacuoles with variably dense cores (*dc*) were found in the DMSO-differentiated cells. *M*: mitochondria. No qualitative changes to neutrophil ultrastructure could be ascribed to nicotine exposure during HL-60 differentiation. The bars represent 1 μm.

The ultrastructural characteristics of HL-60 cells following exposure to nicotine (10^-7 ^-10^-4^M) are shown in Figure 3. While DMSO-induced profound ultrastructural changes over five days in the promyelocytic HL-60 cell line (Figure 3A and 3B), co-exposure to nicotine did not induce qualitatively observable changes in ultrastructure (Figure 3B–D).

### Cell proliferation and viability

Cellular proliferation progressed rapidly over five days in undifferentiated promyelocytes, but was significantly reduced gradually following DMSO induction (reduced cell density and cell viability, as determined by trypan blue exclusion; both *p *< 0.001, *n *= 8, data not shown). However, proliferation of DMSO-induced or -uninduced cells was entirely unaffected by nicotine.

### Nitroblue tetrazolium reduction and expression of CD11b (αM integrin) by HL-60 cells

While DMSO treatment dramatically increased the percentage of HL-60 cells capable of reducing NBT (mean, s.d: 6.1% at baseline; 83.3% at day 5, *n *= 6) and expressing CD11b (mean, s.d: 6.2% at baseline; 88.7% at day 5, *n *= 4) over the five days of differentiation. However, neither terminal differentiation marker was affected by nicotine exposure (data not shown).

### Cellular conformational changes

**Figure 4:**
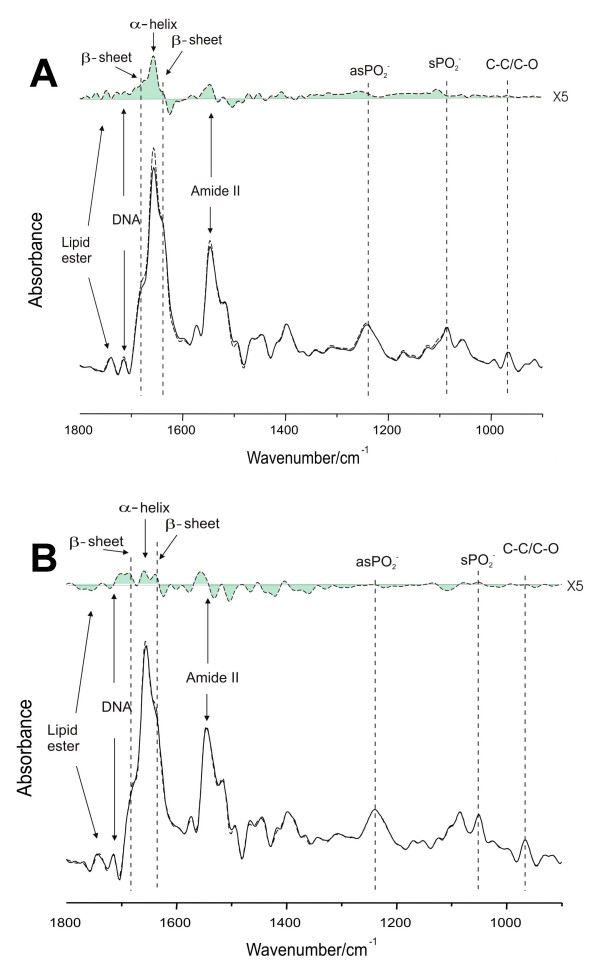
**Characteristic IR spectra of undifferentiated (A) and differentiated (B) HL-60 cells treated with nicotine**. **(A) **Fourier self-deconvoluted (FSD) mean infrared spectra of 5-day HL-60 cells treated with and without nicotine (10^-6^M) (bottom). The difference spectrum between nicotine treated and control HL-60 cells is also shown (top). **(B) **Fourier self-deconvoluted (FSD) mean infrared spectra of 5-day, DMSO-differentiated HL-60 cells treated with and without nicotine (10^-6^M) (bottom). The difference spectrum between nicotine treated and control HL-60 cells is also shown (top).

Fourier-Transform infrared spectroscopy (FT-IR) revealed qualitative alterations to the relative IR band intensities of lipid and DNA signals, and to the ratios of protein (amide I: amide II) and DNA: amide II noted in 5 day cultures of undifferentiated HL-60 cells exposed to nicotine (Figure 4A) and 5 day DMSO-differentiated HL-60 cell cultures (Figure 4B). These specific molecular and sub-molecular profile alterations are indicative of pro-apoptotic changes, as suggested by Zhou et al. [20] who have investigated the IR spectroscopic alterations of apoptotic HL-60 cells subjected to anti-tumor drug induction.

Specifically, Figure 4A shows the mean spectra of HL-60 cells from undifferentiated HL-60 cell cultures (5 days) and those culture cells treated with nicotine after band narrowing by Fourier self-deconvolution (bottom) and the difference spectrum generated by subtracting the mean spectrum of control cells from that of nicotine treated cells (top). After Fourier self-deconvolution, one can readily observe previously overlapping components in the amide I band such as α-helix (1 657 cm^-1^), or parallel and antiparallel β-sheets (1 640 and 1 680 cm^-1 ^respectively). From the difference spectrum that was generated by the integration of three individual IR experiments, one can observe that the contents of α-helix, parallel and antiparallel β-sheets in the undifferentiated HL-60 cell cultures treated with nicotine has increased compared to that in the control group. This observation suggests that the different secondary protein structure in the two groups of mean spectra reflects an altered protein profile in nicotine-treated cells due to pro-apoptotic changes suggested by the increased β-sheets components [20]. Similar changes were found in nicotine treated DMSO-differentiated HL-60 cells, as shown in Figure 4B.

### Cell cycle and apoptosis

While infrared spectroscopy revealed that nicotine induced pro-apoptotic stresses in promyelocytic and DMSO-differentiated HL-60 cells (Figure 4), nicotine did not promote commitment to apoptosis in undifferentiated and DMSO-stimulated HL-60 cells, as determined by flow cytometry. The percentage (mean, s.d.) of apoptotic cells in 5-day promyelocytic cultures were 6.7 (0.4), 6.7 (0.6), and 6.3 (0.4)% for untreated control cells and those treated with nicotine (10^-6 ^M and 10^-4^M), respectively, in four individual experiments. The percentage (mean, s.d.) of apoptotic cells in 5-day DMSO-differentiated cultures were 13.4 (1.1), 14.3 (3.5), and 15.8 (2.0)% for untreated control cells and those treated with nicotine (10^-6 ^M and 10^-4^M), respectively, in six individual assays. Similarly, there were no statistically significant nicotine-induced changes in the mean percentage of cells in the G0/G1 and G2/M growth phases. Likewise, nicotine exerted no statistically significant positive or negative effect on camptothecin-induced apoptosis.

### Nicotine inhibits the PMA-induced oxidative burst and bacterial killing in HL-60 cells

**Figure 5:**
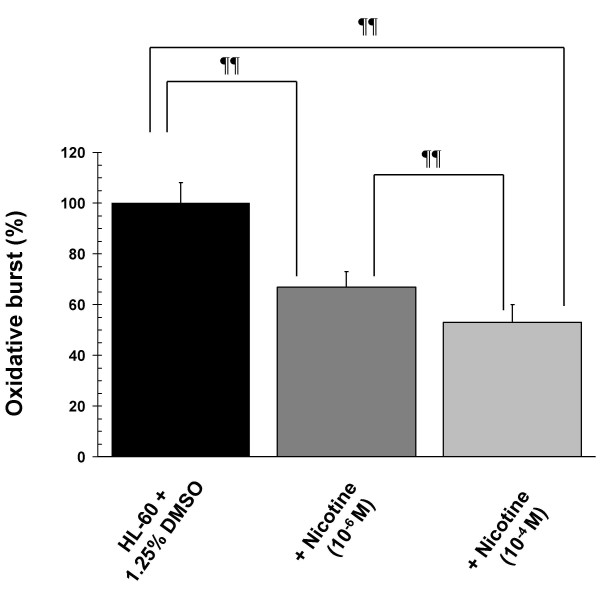
**Nicotine inhibits the oxidative burst in HL-60 cells**. Compared to DMSO-only control cells, the mean oxidative burst (formazan deposition following PMA stimulation) mounted by nicotine-exposed five-day, DMSO-differentiated HL-60 cells was reduced by as much as 48% (*n *= 12, **¶¶ ***p *< 0.001 compared to DMSO-only control cells). Error bars represent standard deviations of the mean.

**Figure 6:**
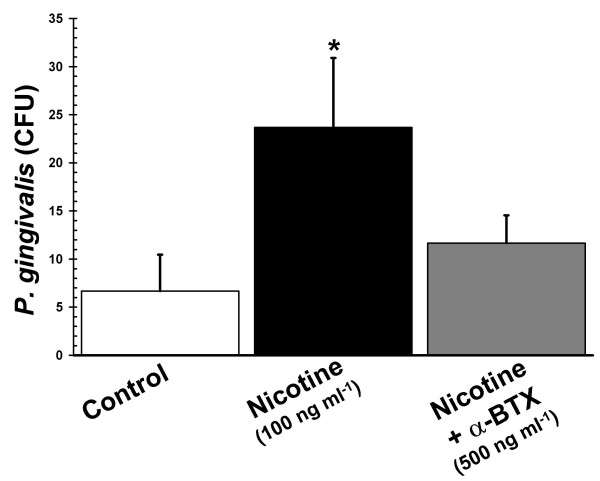
**Nicotine suppresses *Porphyromonas gingivalis *killing in HL-60 cells**. Compared to DMSO-only control cells, nicotine (100 ng ml^-1^)-exposed five-day, DMSO-differentiated HL-60 cells exhibited reduced ability to kill the Gram-negative pathogen *P. gingivalis *(*p *< 0.01). This suppression of bacterial killing was reversed by pre-treating DMSO-induced HL-60 cells with the nAChR-antagonist α-bungarotoxin (α-BTX, 500 ng ml^-1^). Error bars represent standard deviations of the mean, *n *= 3.

Nicotine-exposure during differentiation inhibited the PMA-induced oxidative burst in DMSO-induced HL-60 cells in a dose-dependent manner, as shown in Figure 5. In order to see if a reduced oxidative burst had any functional relevance with respect to bacterial clearance, we performed *P. gingivalis *killing assays. The ability of HL-60 cells exposed to nicotine during differentiation to kill the Gram negative periodontal pathogen, *P. gingivalis*, was compromised compared to unexposed control cells (Figure 6). This nicotine-induced suppression of bacterial killing was inhibited by α-bungarotoxin, suggestingit to be an α7nAChR-dependent phenomenon.

### Nicotine augments the release of MMP-9, but not MMP-2, from HL-60 cells

**Figure 7:**
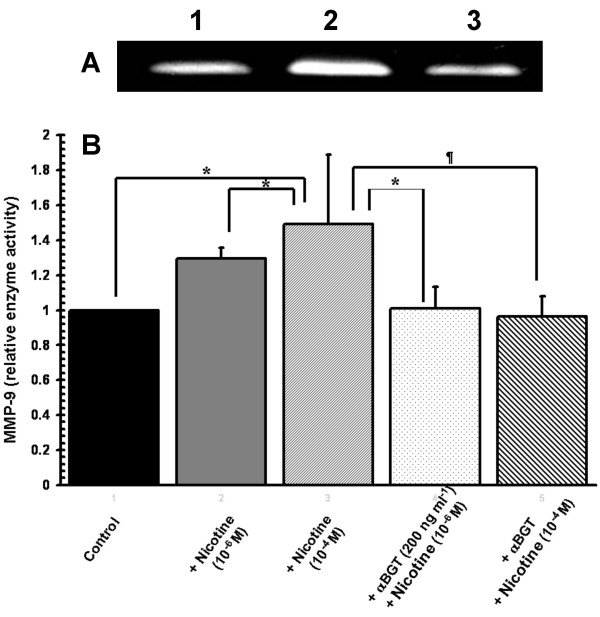
**Nicotine augments LPS-induced pro-MMP-9 release from differentiated HL-60 cells in a dose- and α7nAChR-dependent manner**. **(A) ***Gelatin Zymography*: A representative gelatin zymogram shows increased pro-MMP-9 (92 kDa) activity in the cell-free media (corresponding to 10,000 cells) of five-day DMSO-differentiated HL-60 cells stimulated with LPS (1.0 μg ml^-1^) plus nicotine (10^-4 ^M, Lane 2); compared to LPS alone (Lane 1). Nicotine-induced augmentation of LPS-induced MMP-9 activity is abrogated by pre-incubation of DMSO-differentiated HL-60 cells with an α7-type nAChR inhibitor (α-bungarotoxin, 200 ng ml^-1^, Lane 3). **(B) **Relative fold increases (mean, s.d.) in MMP-9 gelatinolytic activity were quantified by densitometry (***n ***= 5, * *p *< 0.05, **¶***p *< 0.01). Error bars represent standard deviations of the mean.

Nicotine-exposure during differentiation promoted the release of MMP-9 (see Figure 7A and 7B), but not MMP-2, from LPS-stimulated HL-60 cells. The nicotine-induced secretion of MMP-9 was inhibited by α-bungarotoxin, suggesting that this is an α7nAChR-dependent phenomenon (Figure 7B).

### Circulating MMP-9 in human smokers

**Figure 8:**
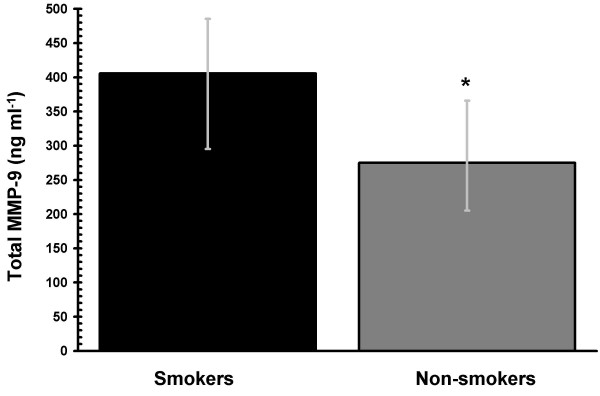
**Tobacco smoking increases the *in vivo *MMP-9 burden**. Total MMP-9 concentrations in the serum of smokers (n = 19) and non-smokers (n = 20) matched for age and gender were measured by ELISA. Circulating total MMP-9 concentrations are significantly increased in smokers compared to non-smokers (* *p *< 0.05). Error bars represent the interquartile range.

Data collected from one female smoker was omitted from the data analyses, as baseline cotinine concentrations did not meet the smoking group criterion (> 20 ng ml^-1^). As expected, median serum cotinine concentrations were significantly higher in smokers compared to non-smokers (239.1 and 0.4 ng ml^-1^, respectively, p < 0.001). In keeping with the *in vitro *HL-60 data, we are able to show that smokers exhibit a significantly higher dose of circulating total MMP-9 than non-smokers *in vivo *(Figure 8).

## Discussion

In addition to neutrophilia, multiple adverse effects of cigarette smoke on mature, circulating neutrophils have been demonstrated. The consequences for neutrophils exposed to tobacco smoke, or to specific tobacco smoke components, include impaired f-actin kinetics and reduced deformability [21], sequestration in the pulmonary microcirculation [22], and activation in the microcirculation [23]. The synthesis of superoxide has been reported to be both increased and decreased in tobacco smoke or nicotine-exposed neutrophils *in vitro *by different research groups. However, such studies have utilized different media for neutrophil suspensions and challenge [6]. Zappacosta et al. [24] have reported that the phagocytic ability of neutrophils pretreated with aqueous smoke extracts is hindered. Importantly, nicotine has been shown to induce the release of elastase from human neutrophils [25], which may play a major role in tobacco-induced destructive diseases, such as COPD. To summarize, while there are certainly conflicting data, it is clear that tobacco smoking profoundly affects multiple functions of circulating and tissue neutrophils and may shift the net balance of neutrophil activities in a destructive direction, as we have recently reviewed [6].

There is a lack of data relating to the effect of tobacco smoke on neutrophil differentiation in humans. It must be acknowledged that our study model, DMSO-differentiated HL-60 cells, are not entirely similar to normal neutrophils. However, this leukemic human cell line does permit the reproducible study of differentiation while retaining many of the key effector functions of primary neutrophils, such as cytokine production, MMP release, generation of an oxidative burst, and the ability to phagocytose and kill microbes. Previously, Armstrong et al. [26] showed that five-day exposure of undifferentiated HL-60 cells to 0.06 and 0.8 μM nicotine induced a significant increase in neutrophil elastase gene activity and protein expression over unstimulated cells, but did not initiate HL-60 cell differentiation. Otherwise, this study is the first, to the best of our knowledge, to examine the effects of nicotine on human neutrophil differentiation. Like Armstrong et al. [26], we noted an increase in cellular protein content by infrared spectroscopy in nicotine-stimulated, undifferentiated HL-60 cells, but nicotine did not induce differentiation in these cells [26]. In rabbits, *in vivo *cigarette smoke exposure has been associated with increased numbers of both mature and band cell (immature) neutrophils liberated from the marrow into the circulation, resulting in preferential sequestration in the microvasculature [2]. Our data shows that exposure to nicotine during differentiation promotes late phase differentiation in human HL-60 cells (Figure 2F).

Nicotine had no effect on cell growth or viability in undifferentiated or differentiating HL-60 cells (Figure 4). Such results are in keeping with the studies of Pabst et al. [12], who have previously observed that nicotine did not affect the viability of mature neutrophils *in vitro*, as determined by trypan blue exclusion. Furthermore, our electron microscopy studies indicate that nicotine does not influence HL-60 cell ultrastructure, relative to untreated control cells.

It has been known for some time that cigarette smoking can induce leukocyte-endothelial adhesion, microvascular and macrovascular entrapment of leukocytes, and leukocyte aggregation in humans and animal models, and that key adhesion molecules including CD11b (a β2-integrin chain) are central to these processes, as we have recently reviewed [27]. We have previously examined the influence of tobacco smoking on CD11b expression, and other adhesion molecules, on circulating human neutrophils *in vivo *[27,28]. We found that tobacco smoking did not acutely influence CD11b expression levels on primary neutrophils. However, Maestrelli et al. [29] have reported that the numbers of neutrophils expressing CD11b and CD18, but not CD11a or CD11c, are increased in chronic smoking subjects with airway obstruction, compared to smokers without airway obstruction, and hypothesized that CD11b/CD18 expression by sputum neutrophils may represent a marker for the development of chronic airway obstruction among smokers. In the current HL-60 model, we have employed CD11b as a marker of terminal differentiation. We noted no statistically significant differences in CD11b expression levels in DMSO-stimulated HL-60 cells exposed to nicotine, compared to unexposed controls. This would imply that, within the limits of this *in vitro *model, any alterations in CD11b expression profiles in chronic smokers are more likely to be the result of exposure of differentiating neutrophils to components of tobacco other than nicotine, or that neutrophil CD11b expression profiles are influenced by smoke components in mature, circulating cells rather than during differentiation. Nicotine did not affect the other maturation marker examined, that is, formazan deposition.

Neutrophils enter the circulation as terminally differentiated cells. Without appropriate inflammatory stimuli, programmed cell death is initiated rapidly. Thus, circulating neutrophils normally have a short life span of less than a day. However, their longevity can be significantly increased in tissues due to the suppression of apoptosis by pro-inflammatory cytokines and other mediators [30]. Induction of apoptosis causes loss of effector function in neutrophils, including chemotaxis, degranulation, generation of the respiratory burst, and is, thus, a key step in the resolution of inflammation. Neutrophils that die by apoptosis are predominantly phagocytosed and destroyed without releasing their proteolytic arsenal [13]. However, neutrophils that die by necrosis release degradative proteases, including MMP-9, and other factors that can contribute to tissue degradation. Consequently, inappropriate suppression of apoptosis would be expected to facilitate or potentiate neutrophil-mediated tissue injury through prolonging neutrophil functional lifespan and abrogating the phagocytotic clearance of dead neutrophils.

The available evidence for an effect of tobacco smoke on neutrophil longevity is controversial. Acrolein, a tobacco smoke component, has been shown to suppress pro-apoptotic signals in systemic neutrophils in an *in vitro *model. Aoshiba et al. [31] reported that nicotine can suppress neutrophil apoptosis. A more recent report concluded high nicotine doses did not influence apoptosis in freshly isolated peripheral neutrophils [32]. Mariggio et al. [33] have presented the alternate message, showing nicotine to be a potent inducer of apoptosis in systemic neutrophils, and Yoshida et al. [32] have reported that high nicotine doses induce DNA cleavage in HL-60 cells. Therefore, an urgent need to clarify the influence of physiologically relevant nicotine exposure on apoptosis in neutrophils has been identified.

We hypothesized that the neutrophilia noted in smokers may partly be due to suppression of apoptosis and that pro-apoptotic signaling may be suppressed even during differentiation. Yet, contradicting this hypothesis, infrared spectroscopy analysis of the molecular profiles of nicotine-stimulated HL-60 cells revealed apoptosis-associated cellular alterations, including increases in the cellular lipid content and in the DNA-protein ratio (Figure 4). However, such pro-apoptotic influences of nicotine on HL-60 cells during differentiation did not result in committed apoptosis, as would have been confirmed by propidium iodide staining in the flow cytometry experiments. Furthermore, nicotine exerted no statistically significant positive or negative effect on camptothecin-induced apoptosis. Clearly, definitive conclusions on the influence of nicotine on apoptosis in differentiating neutrophils would require the detection of biomarkers of cell apoptosis, such as Annexin V staining, Caspase 3 activation, and DNA fragmentation. However, within the limits of our study, nicotine exposure during differentiation neither promoted nor inhibited apoptosis in our *in vitro *assays.

Thus, HL-60 cells are highly resistant to physiologically relevant doses of nicotine during differentiation, with respect to alterations to cell proliferation, viability, cell cycle, ultrastructural characteristics and the development of terminal differentiation markers. However, nicotine exposure during differentiation adversely affects key effector functions in HL-60 cells. It is known that smokers exhibit increased risk of multiple infectious diseases, as discussed earlier. We show that the oxidative burst in response to PMA is quantitatively diminished in HL-60 cells differentiated under the influence of nicotine (Figure 5), which is reflected in an impaired ability to kill the Gram-negative periodontal pathogen, *P. gingivalis *(Figure 6). Interestingly, smokers are known to have increased risk of *P. gingivalis *infections [6,34,35], to harbour greater numbers of *P. gingivalis *cells [6,36] and to be more susceptible to periondontitis than non-smokers [6,37]. It should be noted that the use of a single agent to stimulate ROS production is a study limitation. Nevertheless, further studies into the molecular mechanisms of nicotine-induced suppression of the oxidative burst and bactericidal capabilities in neutrophils, are warranted.

Finally, matrix metalloproteinases are a family of at least eighteen secreted and membrane-bound zinc-endopeptidases. Collectively, these enzymes can degrade most, if not all, the components of the extracellular matrix. Neutrophil-derived, MMP-9-mediated tissue degradation and the instructional role of MMP-9 in directing the inflammatory response are key, and perhaps critical, events in the etiology of chronic obstructive pulmonary disease (COPD) [38]; systemic and cerebral vascular diseases [39], asthma [2,5], and periodontitis[6]. Tobacco smoke exposure is the major environmental risk factor each of these diseases, and herein we show that exposure to the major tobacco constituent, nicotine, during HL-60 differentiation exacerbates the rapid, LPS-induced MMP-9 release in a dose- and α7nAChR-dependent manner (Figure 7). MMP-2 was not similarly influenced. Engagement of α7nAChR on other innate immune cells by nicotine, or the endogenous α7nAChR agonist – acetylcholine, is known to have a profound influence on the inflammatory response to Gram negative stimuli, as we have recently discussed elsewhere [40]. Therefore, these data are in keeping with, and extend, previous reports of increased MMP-9 concentrations in the bronchoalveolar lavage fluids of smoke exposed mice [41], increased neutrophil gelatinase-associated lipocalin [NGAL: a neutrophil-derived protein that associates with MMP-9 [42] concentrations in blood and bronchoalveolar lavage fluid of smokers, compared with non-smokers [43]; and those of Nakamura et al. [44], who were the first to suggest an increased systemic MMP-9 load in smokers. It is not yet clear whether this increase in the circulating MMP-9 burden arises from activation of circulating neutrophils or from translocation from inflammatory cells activated to produce MMP-9 in the pulmonary environment. Further studies into the mechanisms underlying nicotine-induced, α7nAChR-dependent augmentation of LPS-induced MMP-9 release could allow the development of refined and specific therapeutic strategies for the treatment of a number of important tobacco-associated inflammatory diseases and conditions.

## Conclusion

This is the first in depth study of multiple aspects of neutrophil physiology in a consistent cell model and using physiologically relevant nicotine dosing. While HL-60 cells are highly resistant to physiologically relevant doses of nicotine during differentiation, with respect to alterations to cell proliferation, viability, cell cycle, ultrastructural characteristics and the development of terminal differentiation markers, key effector functions are suppressed (oxidative burst and bactericidal capacity) and the release of the MMP-9 is dysregulated. Indeed, an increased circulating MMP-9 concentration in smokers compared to non-smokers is also observed *in vivo*. These findings provide initial mechanistic insights into the increased susceptibility to bacterial infection and inflammation-driven tissue degradation known to occur in nicotine exposed individuals. Furthermore, this study represents a timely and extensive review of the characteristics of the common HL-60 model of leukocyte differentiation.

## Methods

### Materials

HL-60 (CCL-240) and *Porphyromonas gingivalis *(33277) cells were purchased from ATCC (Manassas, VA, USA). Penicillin and streptomycin were purchased from Gibco Laboratories (Grand Island, NY, USA). Fetal bovine serum and Hema-3 staining kits were from Fisher Scientific (Nepean, ON, Canada). Bromophenol blue, CaCl_2_, cotinine, Escherichia coli K12 LPS, gelatin, gentamicin, Giemsa-Wright, metronidazole, NaCl, nicotine, nitroblue tetrazolium (NBT), paraformaldehyde, phorbol 12-myristate 13-acetate (PMA), RPMI 1640 medium, Triton X-100, and trypan blue were bought from Sigma-Aldrich (St. Louis, MO, USA). Glacial acetic acid, glycerol, and methanol were supplied by Fisher Scientific (Ottawa, ON, Canada). OsO4, Epon, lead citrate/uranyl acetate were bought from Electron Microscopy Sciences (Hatfield, PA, USA). Anti-α7 nicotinic acetylcholine receptor antibodies (AChRα7 [H-302]), HRP- or FITC-conjugated goat anti-rabbit IgG secondary antibodies, and α7 protein positive controls (mouse frontal brain extractions) were purchased from Santa Cruz Biotechnology Inc. (Santa Cruz, CA, USA). Nitrocellulose membranes were from BioRad Laboratories (Mississauga, Canada). Amersham Biosciences Corp (Piscataway, NJ, USA) enhanced chemiluminescence (ECL) western detection kits and x-ray films were used for visualization of western blots. PE-conjugated anti-CD11b (clone ICRF44) and Mouse IgG1, K (MOPC-21) isotype control antibodies were purchased from BD Biosciences Pharmingen (San Diego, CA, USA) and Sigma-Aldrich (St. Louis, MO, USA), respectively. Matrix metalloproteinase (MMP)-2, MMP-9, and Quantikine human MMP-9 (total) immunoassays were bought from R and D Systems (Minneapolis, MN, USA). Gifu anaerobic medium (GAM) broth was obtained from Nissui Pharmaceutical Co., Ltd, Tokyo, Japan.

### Subjects

The study population has been previously described [45]. Briefly, twenty smokers (10 female/10 male; 33.8, s.d. 8.8 years) and twenty age (32.6, s.d. 9.1 years) and gender matched non-smokers were recruited from among the patients and staff of Guy's, King's and St. Thomas' Dental Institute and King's College London. Smokers were required to report consumption of ≥ 10 cigarettes per day, whereas age and gender-matched non-smokers were required not to have smoked for ≥ 3 years. A medical history was taken for each subject and those with diabetes, pregnancy, on anti-inflammatory drug regimens including aspirin and other NSAID's, and with any history of chronic inflammatory disease or leukocyte dysfunction were excluded. Ethical approval was granted by the local Health Research Ethics Board, and written, informed consent was obtained from each subject. Serum samples from each subject were stored at -80°C until required.

### Growth of HL-60 cells

HL-60 (ATCC CCL-240) cells were cultured in RPMI 1640 medium with 0.3 g dm^-3 ^*L*-glutamine and 2.0 g dm^-3 ^sodium bicarbonate supplemented with 10% (v/v) heat-inactivated fetal calf serum, penicillin (50 U ml^-1^) and streptomycin (50 μg ml^-1^). The cells were cultured at 37°C in an atmosphere of 5% CO_2 _and 100% humidity.

### Expression of nicotine receptors by HL-60 cells

Total cell extracts (40 μg of protein) were electrophoresed through 10% PAGE gels and transferred onto nitrocellulose membranes (0.45 μm). Membranes were blocked with 5% milk casein. A primary polyclonal antibody (AChRα7 [H-302], 1:200 dilution in 3% milk casein) recognizing amino acids 367–502 mapping to the C terminus of α-7 subunit of the human receptor. The secondary antibody (HRP-conjugated goat anti-rabbit IgG) was used at a dilution of 1:1000 in 3% milk casein. Binding sites were visualized by ECL and application to X-ray film. Quantitation was done using Quantiscan v3.0 (Biosoft, Cambridge, UK) with background set to correct as an interpolated minimum. α7AChRs were also visualized by fluorescent immunocytochemistry using primary antibody (AChRα7 [H-302]), a FITC-conjugated secondary anti-rabbit IgG antibody, an inverted fluoresence microscopic (Nikon Eclipse TS100 with Nikon Plan Fluor objectives and Omega Optical Fluor filter set) and an inverted confocal microscopic (Olympus IX70 with Nomarski DIC optics). Images were captured by InCytIm 1 software (Intracellular Imaging, Cincinnati, OH) and FluoView software (Olympus), respectively. Control cells were treated similarly, but without the primary antibodies.

### Differentiation of HL-60 cells

Granulocytic differentiation was initiated by addition of 1.25% DMSO (final concentration) for 5 days with or without various doses of nicotine (10^-7 ^to 10^-4 ^M). Multiple aspects of neutrophil differentiation were monitored. Gross morphological examination of the HL-60 cells was performed microscopically following Hema-3 staining, with ultrastructure characteristics examined by electron microscopy. For electron microscopy, cells were fixed in 4% paraformaldehyde, postfixed in OsO4, dehydrated, embedded in Epon, cut into thin sections, stained with lead citrate/uranyl acetate and viewed on a Philips transmission CM-10 electron microscope (Philips Electronics, Eindhoven, Netherlands). The ability to reduce NBT (1.0 mg ml^-1^) after incubation with phorbol 12-myristate 13-acetate (PMA, 200 ng ml^-1^) for 30 mins, 37°C was determined by microscopically evaluating formazan deposition in 200 randomly selected cells. The percentage of cells in which formation of intracellular formazan deposits occurs increases with maturation [46]. Cell density was measured using a hemocytometer. Viability was determined by 0.4% trypan blue staining. Expression of the terminal differentiation marker CD11b was determined by flow cytometry, as described below. Finally, the re-distribution of key cellular components during differentiation was determined by infrared spectroscopy.

### Expression of CD11b (integrin αM) by HL-60 cells

Cells were stained with PE-labeled anti-CD11b monoclonal antibody (ICRF44 at 20 μl per 1 million cells in 100 μl total staining volume). An isotype-matched antibody (Mouse IgG1) was used as a negative control. Data from 10000 cells were collected using an EPICS Altra-fluorescence-activated cell sorter (Beckton Dickinson, San Jose, CA, USA).

### Conformational changes in differentiating HL-60 cells

FT-IR spectroscopy can monitor structural alterations to key cellular components, including membranes, proteins, and nucleic acids. Therefore, conformational changes in nicotine exposed differentiating HL-60 cells or unexposed cells were examined by FT-IR spectroscopy, as described previously [47]. Essentially, HL-60 cells were washed in 0.9% NaCl, loaded onto BaF_2 _windows and vacuum-dried (25 Torr). Infrared spectra were recorded using a Bio-Rad FTS-40A IR spectrometer (Bio-Rad Laboratories) at a nominal resolution of 2 cm^-1^. Two separate films from each subject sample were measured, each spectrum consisting of 256 co-added interferograms, apodized with a triangular smoothing function before Fourier transformation. All IR spectra were baseline corrected and area normalized between 900 – 1800 cm^-1 ^using WIN-IR software (Bio-Rad Laboratories, Cambridge, MA) and analyzed using WIN-IR and in-house FT-IR spectroscopy software (Molecular Spectroscopy Group, National Research Council, Winnipeg, Canada). Alterations to DNA were determined by qualitative analysis of the DNA-specific spectral bands at 965 cm^-1 ^(C-C/C-O stretching vibration), 1087 cm^-1 ^(*v*_s_PO_2_^- ^stretching vibrations), 1240 cm^-1 ^(*v*_as_PO_2_^- ^stretching vibrations), and 1713 cm^-1^(characteristic of base-paired DNA strands). Qualitative lipid changes were assessed by analysis of the spectral band at 1740 cm^-1^originating from lipid ester C = O group. Qualitative membrane protein changes were assessed by analysis of the spectral components in the amide I band arising from the amide C = O stretching vibration of the peptide groups in all proteins such as α-helix (1 657 cm^-1^), or parallel and antiparallel β-sheets (1640 and 1680 cm^-1 ^respectively).

### Apoptosis assays

The percentage of apoptotic cells in control and nicotine-treated (10^-4^M), differentiated (5 day), HL-60 cells was determined by propidium iodine (PI) DNA staining. Briefly, 3.0 × 10^6 ^cells were washed in PBS, 4°C, centrifuged (200 × *g*, 5 min, 4°C), and resuspended in 0.5 ml of cold PBS. Cells were fixed in ice cold 70% ethanol. Fixed cells were washed in PBS, centrifuged (300 × *g*, 5 min, 4°C) and resuspended in cold PBS with 100 U ml^-1 ^RNase A and incubated at room temperature for 30 min. The cells were further incubated in 25 μg ml^-1 ^PI (final concentration, 30 min, room temperature) in the dark. PI staining was analyzed by flow cytometery (EPICS Altra-Fluroscence-Activated Cell Sorter). The ability of nicotine to block camptothecin (10 μM)-induced apoptosis in differentiated (5 day) HL-60 cells was also examined.

### Quantification of the oxidative burst

The ability of HL-60 cells, differentiated for 5 days in the presence or absence of nicotine or cotinine, to mount an oxidative burst in response to PMA was quantified colorimetrically. 2 × 10^6 ^cells were treated for 1 h with 0.5 mg ml^-1 ^NBT and 200 ng ml^-1 ^PMA (both final concentration), at 37°C and the reaction stopped by adding 100% acetic acid. After removal of supernatant (12000 × ***g***, 2 mins, RT), any formazan in the cell pellet was extracted using 50% acetic acid and sonication. Cellular debris was pelleted by centrifugation (12 000 × ***g***, 5 mins, RT) and reduced NBT quantified colorimetrically at 560 nm using a Bio-Rad model 550 microplate reader (Bio-Rad Laboratories).

### Intracellular bacterial survival assay

*Porphyromonas gingivalis *cultures were grown anaerobically in Gifu anaerobic medium (GAM) broth in a Coy AALC anaerobic chamber (Coy Laboratory Products, Grass Lake, MI, USA). Treated (nicotine differentiated) and control 5-day, DMSO-differentiated HL-60 cells were incubated at 37°C with *P. gingivalis *for one hour (MOI = 10). Extracellular, non-adherent bacteria were removed by washing, while extracellular adherent bacteria were killed by addition of gentamicin (300 μg ml^-1^) and metronidazole (200 μg ml^-1^) for one hour. After washing, internalized bacteria were released by lysis of the mammalian cells in sterile distilled water for 20 min, a treatment that does not affect bacterial viability. Serial dilutions of the lysates were than plated on GAM and cultured anaerobically for CFU enumeration.

### Matrix Metalloproteinase (MMP) secretion

Five-day DMSO-differentiated (with or without various doses of nicotine (10^-7 ^to 10^-4 ^M) were stimulated with 1.0 μg ml^-1 ^(final concentration) purified LPS for 1 or 24 hr and cell-free supernatants collected. The importance of the α7 nAChR in nicotine-induced MMP-9 release was determined by using the α7 nAChR antagonist α-BTX (200 ng ml^-1^). MMP secretion was measured by gelatin zymography and densitometry. Briefly, 20 μl aliquots of cell-free supernatant (corresponding to 10000 cells) were mixed with 10 μl of zymogram sample buffer (62.5 mM Tris-HCl pH 6.8, 25% Glycerol, 4% SDS, and 0.01 % Bromophenol Blue) and run on 8.0% SDS-polyacrylamide gels complemented with 0.1% gelatin type I. SDS was removed from the gels by washing twice with 2.5% Triton X-100, 30 min, RT, allowing the separated proteins to renature. Subsequently, the gels were immersed into TCS buffer (50 mM Tris-HCl, pH 7.4, 0.2 M NaCl, 5 mM CaCl_2_) and incubated for 18–24 hr, 37°C then stained with 0.25% (w/v) Coomassie Blue, 60 min, RT. Active MMP-9 and MMP-2 were noted as clear bands against the blue background after destaining (30 mins, RT, in 10% glacial acetic acid and 30% methanol). MMP-9 and MMP-2 bands were quantified by densitometry, as described earlier.

### Quantification of serum cotinine

Serum cotinine levels were determined by gas-liquid chromatography, as previously described [48]. Smokers can be reliably differentiated from non-smokers by serum cotinine concentration at an optimal cut-off point of 13.7 ng ml^-1 ^[17]. Accordingly, smokers and non-smokers were required to present with serum cotinine levels of = 20 ng ml^-1 ^and < 13.7 ng ml-1, respectively.

### Circulating MMP-9 in smokers and non-smokers

The circulating concentrations of total MMP-9 were determined in smokers and non-smokers by Quantikine ELISA, according to the manufacturer's instructions.

### Statistical analyses

Where appropriate, statistical analysis was done by post hoc application of Duncan's New Multiple Range Test, assuming a significant difference at p < 0.05. Regression lines were fitted to the raw data using SigmaPlot v7.101 (SPSS Incorporated, Chicago, ILL). Non-parametric tests (Mann-Whitney U) were required for analyses of in vivo cotinine and MMP-9 concentrations.

## Authors' contributions

DAS conceived of the study. MX, HRB and DER carried out most of the experiments reported in the manuscript. MX and KZL designed and carried out the infrared spectroscopy experiments and analyzed the infrared data. JES performed the statistical analyses. DAS, MX, ASG, JES and KZL participated in the study design and coordination. DAS drafted the manuscript with input from all authors. All authors read and approved the final manuscript.
